# Enforcement of the Use of Digital Contact-Tracing Apps in a Common Law Jurisdiction

**DOI:** 10.3390/healthcare10091613

**Published:** 2022-08-25

**Authors:** Tsz Ho Kwan

**Affiliations:** Stanley Ho Centre for Emerging Infectious Diseases, The Chinese University of Hong Kong, Hong Kong 999077, China; thkwan@cuhk.edu.hk

**Keywords:** digital contact tracing, non-pharmaceutical intervention, public health law, COVID-19

## Abstract

Digital contact-tracing systems have been widely implemented worldwide with different system designs and implementation policies for the purpose of tracking potentially exposed individuals. The use of a digital contact-tracing app in Hong Kong has been mandated for visiting certain premises by legislations. This paper reviewed the regulations promulgated specifically for the prevention and control of COVID-19 and identified those associated with the digital contact-tracing system. A comprehensive search in newspaper databases was performed to explore the enforcement of the mandated use of the digital contact-tracing app. The three facets of regulations in relation to digital contact tracing were examined: duty to disclose information, requirements and directions to businesses, and compulsory testing. The use of digital contact-tracing data for non-public health purposes was also reported. Our analyses showed that prosecution of non-use or the use of fraudulent digital contact-tracing apps was not limited to COVID-19-specific legislations. The flexible approach ensured the enforcement of the use of the digital contact-tracing app, but the judiciary’s test must be passed in future cases.

## 1. Introduction

Since the SARS-CoV-2 virus being brought into Hong Kong, over 16% of the population have been infected [1]. The epidemic curve of local transmissions of the virus could be characteristically described by distinct epidemic waves. The first wave between January and June 2020 causing 1128 infections resulted in the enactment of specific epidemic control legislations [2]. These initial statutory measures included mask-wearing, prohibition of group gatherings, and inbound travel restrictions and compulsory quarantine. In this wave, the entertainment exposure setting contributed to onward transmissions and ensuing transmission cluster formation. The larger second wave, which broke out following the first in the same year, consisted of over 400 transmission clusters [3]. It was found that the transmission speed in exposure settings relating to daily activities was particularly high in this wave. While social activities tended to cause another cluster in other social activities, the transmission chain normally ended within the households. To curtail transmissions, more stringent non-pharmaceutical interventions, such as limiting the operating hours and number of customers of restaurants, and closure of entertainment and personalized service premises, were enforced by legislation [4]. These policies were able to minimise transmissions in the targeted exposure settings, but infection risks in other unrestricted exposure settings materialised. The most notable example was that the closure of public entertainment premises led to the emergence of private gatherings/parties [5]. Workplace transmission was also prevalent as the work-from-home arrangement had always been a suggestion rather than a rule. These measures were amended from time to time since then according to the latest development of the pandemic and the availability of vaccines [6]. A digital contact-tracing mobile app named LeaveHomeSafe was subsequently launched by the Hong Kong government in November 2020. The use of the app was initially voluntary until late 2021 when its use became compulsory when entering into certain types of venues, such as government buildings, restaurants, and entertainment premises [7]. 

The check-in-based digital contact-tracing system in Hong Kong has been described previously [8]. In brief, at the entrance of the premises where check-in using the digital contact-tracing app was necessary, a notice with a venue-specific quick response (QR) code would be posted. The user would be asked to scan the QR code using their app and show the screen to the premise manager for confirmation. One could click the “leave” button to record the leaving time once left. These records would only be saved in the user’s phone, unless they were later diagnosed with COVID-19, when they would be asked to upload the history of their whereabouts to the server. These patient histories would be constantly pulled by other users’ mobile phones, and comparison with their own histories would be conducted locally [9]. A notification would be issued if the user were in the same venue with a patient for at least a second or took the same taxi within 24 h after a patient did. This is referred to as a decentralised digital contact-tracing system. 

In public health, prevention of a health risk can be characterised into three levels, namely primary, secondary, and tertiary. In terms of prevention of an infectious disease, primary prevention refers to the prevention of acquiring the pathogen causing an infection; secondary prevention aims to prevent the development of diseases after infection had occurred; tertiary prevention focuses on preventing complications, morbidity and mortality for established diseases. The digital contact-tracing system could play a major role in secondary prevention, where exposed individuals could receive a notification so that they could comply with the regulations at the material time for, inter alia, undergoing testing and quarantine [10]. By identifying, isolating, and treating infected individuals, the transmission risk in the population could be minimised (primary prevention) [11], and the risk and severity of disease development could be reduced by early treatment (tertiary prevention). Thus, a digital contact-tracing system could become a gateway to all three levels of prevention. 

However, similar to other non-pharmaceutical or social distancing interventions, the enforcement of these measures by legislation inevitably restricts citizens’ freedoms and rights [12]. These restrictions could be classified into three dimensions: assembly restrictions, movement restrictions, and privacy restrictions [13]. Closure of schools and workplaces, cancellation of public events, and limiting the size of gatherings were considered to restrict assembly, so as to reduce the risk of direct transmission between individuals. By not operating public transport, imposing a stay-at-home policy, and restricting domestic and international travel, the freedom of movement was limited to avoid the introduction and transmission of the pathogen. Contact tracing, even without restricting one’s freedom of movement, limits citizens’ right to privacy when being asked about their daily activities, contacts, geographical trajectories and medical histories, which could be personal [14]. Hong Kong is a signatory party to the International Covenant on Civil and Political Rights (ICCPR) and the International Covenant on Economic, Social and Cultural Rights (ICESCR). Both covenants were signed by the United Kingdom (UK) when Hong Kong was its dependent territory before the handover in 1997. Fundamental rights, including, among other things, life, privacy, and freedom of movement, are protected by ICCPR. These rights are not always absolute and non-derogable. In fact, most are restrictable within limits. Among the list of legitimate grounds for limiting or derogating human rights, public health is one of the examples and has been stipulated in the Convention [15]. Separately, the ICESCR protects essential living rights in a modern society, including rights to health, education, work, and “an adequate standard of living” including food and shelter. Signatory parties owed a duty to take steps to realise protection of these rights, such as by administrative means or legislations (Chan Noi Heung v Chief Executive-in-Council, 2006). It has long been established that a dualist system has been adopted in Hong Kong (GA and others v Director of Immigration, 2014) where international laws must be transposed into local legislations before they can be directly applied (Ubamaka v Secretary for Security, 2012). The ICCPR was transposed as the Hong Kong Bill of Rights Ordinance (BORO) in 1990. However, the ICESCR has never been transposed. After handover, both treaties have remained in force “as applied to Hong Kong” as prescribed by the Hong Kong Basic Law (HKBL). Although the Standing Committee of the National People’s Congress nullified three sections in the BORO later, the protection of ICCPR rights largely remained unchanged and effective [16]. In addition, the HKBL also has separate provisions, offering parallel human rights protection in, for example, Articles 24 to 41.

Against these backgrounds, this study aimed to examine the enforcement of the digital contact-tracing system in Hong Kong, a jurisdiction where common law applies. 

## 2. Materials and Methods

This study concerned two research questions. The first one was the legal foundations as to the mandated use of the digital contact-tracing app. Then, we examined how it was enforced by looking at cases in real life of whether or not the purpose of its use was initially intended. The answers to these questions would lead us to the future of the digital contact-tracing system. 

After the global pandemic of severe acute respiratory syndrome (SARS), the World Health Organization (WHO) revised the International Health Regulations (IHR) [17] in 2005, which is a legally binding instrument on all WHO Member States, including China, of which Hong Kong is its inalienable part (Art. 1 HKBL). Member States’ development of a legal framework for infectious disease prevention and control without unnecessary hindrance to international mobility is required by the IHR. It is highlighted that “human rights and fundamental freedoms” have to be respected (Art. 3(1) IHR). As a result, a bill was passed in Hong Kong in 2008, which then became the Prevention and Control of Disease Ordinance (Cap. 599). In view of the emerging situation in Hong Kong, the Chief Executive-in-Council enacted public health emergency regulations with the power conferred under Cap. 599. In this paper, these regulations will be reviewed, and those relevant to the enforcement of (digital) contact tracing will be analysed using a doctrinal approach [18].

As the implementation of digital contact tracing in Hong Kong was a controversial topic, incidences relating to it were often reported by the news media. Separately, there were no data released by the government about the enforcement of digital contact tracing. Therefore, publicly available news reports, online or printed, could be valuable in the investigation process, as used previously [19]. Supplementing the doctrinal analysis, the second part of the assessment would adopt a socio-legal approach to assess its enforcement in the social context. A comprehensive search was conducted on Google News and Wisenews. The latter was a newspaper archive database collecting online and printed news articles in mainland China, Hong Kong, Macau, and Taiwan. The list of keywords used in the search was: “LeaveHomeSafe” (the name of the digital contact-tracing app used in Hong Kong), “on1sam1ceot1hang4” (the Chinese name of the app), and “on1sam1” (the first two characters of the app name, as an abbreviated title). The search period was between 1 November 2021, the month in which the app was launched, and 24 June 2022. As some news agencies had ceased operation during the search period and the websites had been taken down, additional searching was conducted using Google News and the search engine when a related item was not archived by the agency or the newspaper database but by other people. 

## 3. Results

Subsidiary legislations under Cap. 599 ranged from A to L, of which C onwards were enacted due to the COVID-19 pandemic. Cap. 599A was amended after the pandemic and it covered a wide range of disease prevention and control measures, including: (1) notification of infectious diseases, (2) disease prevention, medical surveillance, examination and test, (3) vaccination and prophylaxis, (4) quarantine and isolation, (5) criminalising exposure of the public to infection, (6) disease control measures such as restrictions on premises or conveyance and others, (7) control of laboratories’ handling of scheduled infectious agents, (8) declaration and certification of cross-boundary conveyances, (9) pratique, (10) restrictions on landing and departure of cross-boundary aircrafts, and (11) prohibition from leaving Hong Kong. This subsidiary legislation was commonly used for issuing quarantine and isolation orders to exposed and infected individuals, respectively. It was also used to isolate building blocks for compulsory testing (s. 25). Cap. 599B was related to the supply of water to boats and wharves. In terms of international travel, Cap. 599C and Cap. 599E were the legal foundations for compulsory quarantine of inbound travellers, and Cap. 599H regulated inbound conveyances. Social distancing measures and other preventive measures included: Cap. 599G on the restriction of gatherings in public and/or private premises, Cap. 599I on mandatory mask wearing in public places, Cap. 599K on the authorisation of the use of non-registered COVID-19 vaccines, and Cap. 599L on the restriction of unvaccinated or not fully vaccinated persons from entering into certain premises or public transport carriers. The relevant regulations in relation to the use of the digital contact-tracing system included Cap. 599D on information disclosure, Cap. 599F on business operation directions, and Cap. 599J on compulsory testing. 

Cap. 599D imposed a duty on a person who was in possession of information or who had information under their control, to provide such information to identify and trace potentially exposed individuals. In 2021, two persons were convicted of an offence contrary to s. 3(4) of Cap. 599D for giving false or misleading information in relation to their whereabouts during the incubation period after arriving to Hong Kong (KCCC 1302/2021). One of the defendants introduced the alpha variant into Hong Kong and later triggered two rounds of mandatory testing for foreign domestic helpers [20]. The Magistrate in this case emphasised in her judgement that the sentence was not meant to punish infected individuals but to send a message to the public that the regulations have to be complied with for the prevention of transmission. The definition of “information” under this ordinance included “places where the person has been to”, “the medical history of the person”, and “any contact between the person and other persons” (s. 4(3) Cap. 599D). Although at that time the digital contact-tracing app had not been developed, it is likely that, if someone refused to provide their digital contact-tracing data for delineating their whereabouts, they would be similarly prosecuted under Section 3 of the ordinance.

Cap. 599F empowered the Secretary for Food and Health to lay down requirements and directions to businesses, primarily in catering and entertainment premises. These directions normally included operating hours, maximum number of patrons allowed, and number of persons per table. The use of the digital contact-tracing app, among other things, has been made mandatory by the requirements set forth similarly. Customers must log their location using the app if they visit restaurants, bars, entertainment premises, hotels, shopping malls, department stores, markets, supermarkets, and some other places. The manager of the premises must ensure compliance, or they and the incompliant customers would be liable for an offence. In early 2022, some customers did not use the app in a catering banquet and they, and the restaurant, were fined [21]. 

There were a few occasions where a fraudulent digital contact-tracing app was involved. The first case happened in October 2021 when the defendant was alleged to use a fraudulent app in a restaurant (KCCC 3302/2021) [22]; she was later charged with using a false instrument contrary to s. 73 Cap. 200. The charge was subsequently dropped [23]. Two cases that are more recent were prosecuted as an offence of knowingly misleading a police officer, contrary to s. 64 Cap. 231. On the other hand, the use of a fraudulent digital contact-tracing app for granting access to government buildings could be considered as trespassing in tenement under the control of any department contrary to s. 4(3) Cap. 228 (ESCC 593/2022) [24]. It is worth noting that in this case, the defendants were initially charged with using a false instrument but ended up with a trespass offence [25]. 

Cap. 599J was the key legislation for imposing compulsory testing and related entry/exit restriction. The digital contact-tracing system serves an ancillary role of notifying potentially exposed individuals who were in a place where a patient had visited, advising them to undergo compulsory testing. A compulsory testing notice could, however, not be sent through the app if the exposed location was not covered by the mandated use of digital contact tracing. The first year of implementation of the digital contact-tracing system resulted in over 10,000 notifications and 320,000 tests [26]. 

The use of the digital contact-tracing app was not limited to the notification of potential exposure. In line with the aim to prevent and control diseases, digital contact-tracing data were leveraged as evidence of deliberately exposing others to the infection, contrary to s. 32(1) Cap. 599A. Two individuals were arrested for visiting multiple places after receiving a positive test result [27]. These data had also been used in criminal investigations unrelated to public health. In September 2021, it was reported that the police checked escaping individuals’ digital contact-tracing records to confirm the presence of witnesses in a case of assault occasioning actual bodily harm [28]. In June 2022, there was a shooting incident in Central, and it was reported that the digital contact-tracing data were collected to trace the perpetrators [29]. In another case, the defendant had been granted bail on the condition of a curfew, but he breached the bail condition by being found in a park after 11pm (DCCC 771/2020). In his defence, he claimed that he was chatting with friends in the park after dinner and hence lost track of the time. The Principal Magistrate in that case demanded the records of the digital contact-tracing app. The defendant could not provide such records and thus the bail was refused. These cases illustrated that the data collected by the digital contact-tracing app could be accessed or be requested to be made available to a third party, whether or not they were “authorized officers” defined in, for example, s. 2 Cap. 599F, for non-public health purposes.

## 4. Discussion

Privacy issues have always arisen from the implementation of a digital contact-tracing system [30]. A balance has to be carefully struck between protecting public health and privacy [31]. By adopting a decentralised approach, the user retains the authority to their data, and their right to privacy could be protected. However, under the duty imposed by Cap. 599D, such a right would be limited if they were diagnosed with COVID-19. In Australia, the government primarily adopted a decentralised system but with centralised identifiers. The COVIDSafe Act, being an amendment to the Privacy Act, was passed to protect the rights of users and restrict the use of the app. The Act emphasised the voluntary nature of the use of the digital contact-tracing app, limited the legal purpose of the use of app data, made any compulsory use illegal, and stipulated a sunset condition for the entire system [32]. A similar view was shared in [31] that these measures should only be temporary and not be extended to the post-COVID-19 period. The user should also be informed of, at the time of data collection, the duration of retention, the purpose of data collection, and the entity who could access the data [33]. In contrary, despite earlier assurances, Singapore confirmed that digital contact-tracing data would be used for criminal investigation [34], which was beyond the initial purpose of data collection. The latter was akin to the situation in Hong Kong in which digital contact-tracing data would primarily be used for tracking exposed individuals; they would and were also used for criminal investigation when needed in the absence of similar provisions in the COVIDSafe Act. In Indonesia, a further discussion on disclosing patients’ health records was noted [35]. Citing the pandemic as a “special condition”, the right to privacy, including the common law duty of confidentiality [36], could be encroached. Policies protecting the privacy in relation to the use of such digital contact-tracing apps had a primary purpose of building trust between the government and the users. The potential concerns about how the data would be handled could form a negative feedback loop, causing distrust in the government and impeding the uptake of the digital contact-tracing app [37]. Additional measures to enforce the (voluntary) use of the app would be needed. It is noted, however, that criminal measures may not necessarily support public policies, but conversely undermine trust and create stigma [38].

The use of the digital contact-tracing app in Hong Kong has been made compulsory in many premises involving daily activities. People who have been sceptical about the collection of personal information with the app tried to use alternative measures to avoid the mandate. It appeared that prosecution turned away from “using a copy of a false instrument” as the charge. Although the reason was unknown to the public, it is worth noting that the definition of “false instrument” has recently been reviewed in a Court of Final Appeal case (HKSAR v Chan Kam Ching). The law enforcement agencies and the Department of Justice were willing to look into various areas of criminal laws to identify suitable charges. In cases where the police were involved in the inspection process, the most likely offence alleged would be knowingly misleading a police officer (s. 64 Cap. 231).

Some may argue that the mandate of digital contact-tracing app and vaccination in certain premises would restrict the freedom of movement and right to privacy. In Law Yee Mei v Chief Executive of Hong Kong SAR and others (2022), HKCFI 688, Justice Coleman reiterated that these ICCPR rights were derogable subject to the proportionality test, and that the ICESCR “does not have the force of law in Hong Kong” without local legislations. This provided the legal foundation for the implementation of a digital contact-tracing app for the purpose of prevention and control of COVID-19. However, although rights in the ICESCR cannot directly be relied upon, a residual power is left to the court to ensure its implementation [39], and the covenant should enjoy a constitutional status in Hong Kong [40]. Therefore, in Clean Air Foundation Ltd. v Government of the HKSAR (HCAL 35/2007), it was held that the government owed a duty to take steps to protect the right to health, which was a right protected by the ICESCR. Nevertheless, even if it were enforceable in Hong Kong, the challenge would likely fail as the limitation of rights was likely to be found proportionate.

The landscape of the pandemic has changed in the past year. Some countries relaxed social distancing measures and some even eased border restrictions [41]. The UK stopped the mandatory use of digital contact-tracing apps in February 2022 as the virus became endemic. During the peak of the pandemic in Hong Kong in early March 2022, the digital contact-tracing app no longer issued exposure notifications due to the overwhelming case load [42]. It only resumed later that month when the number of cases went down to under 5000 [43]. With the zero-COVID-19 policy, the implementation of the mandated use of the digital contact-tracing app and ensuing controversies would likely remain. Transparency, trust building, and public education on both highlighting its public health necessity and addressing the privacy concerns are paramount in promoting its uptake [44,45]. Reliance on punitive measures, while they could be effective in enforcing the use of digital contact-tracing app, may erode trust between citizens and the government.

We believe that this paper, as the first of its kind in summarising the legislations related to the use of a digital contact-tracing app and its enforcement in real life in Hong Kong, a common-law jurisdiction, provides a useful contribution to the literature and health-policy design. We found that no specific legislations were designed to limit the use of the digital contact-tracing app, unlike in some other countries such as Australia. The mandate was in a more flexible form of specifications and directions issued by the Secretary for Health. The prosecution was, therefore, willing to consider a variety of legal provisions, even outside the scope of the dedicated prevention and control of disease ordinance, to enforce the use of the digital contact-tracing app when necessary. Judgements laid down by the higher courts would clear up the uncertainties in the enforcement and prosecution process.
